# Mebendazole-Induced Blood-Testis Barrier Injury in Mice Testes by Disrupting Microtubules in Addition to Triggering Programmed Cell Death

**DOI:** 10.3390/ijms23084220

**Published:** 2022-04-11

**Authors:** Mingqian Huang, Chang Wang, Ying Yao, Huiling Li, Yejin Yao, Yunfei Zhu, Yiqiang Cui, Yan Yuan, Jiahao Sha

**Affiliations:** Department of Histology and Embryology, Nanjing Medical University, Nanjing 210000, China; huangmq0515@outlook.com (M.H.); wangchangnjmu@126.com (C.W.); yaoyimg@163.com (Y.Y.); lihuiling@njmu.edu.cn (H.L.); yyj_19960608@163.com (Y.Y.); zhuyunfei@njmu.edu.cn (Y.Z.); cuiyiqang@126.com (Y.C.)

**Keywords:** blood-testis barrier, Sertoli, mebendazole, tubulin, apoptosis

## Abstract

Mebendazole (MBZ) is a synthetic benzimidazole known for its antiparasitic properties. In recent years, growing evidence showed that MBZ was also used as an anti-tumor agent. However, whether (and to what extent) this drug treatment affected the male reproductive system was not well-understood. In this study, male C57BL/6 mice were injected with 40 mg/kg/day of MBZ. The treatment was for 3 and 7 days. Our results showed that the injected mice exhibited an abnormal spermatogenic phase with a significant decrease in sperm. We further detected microtubule disruption and transient functional destruction of the blood–testes barrier (BTB) in the MBZ-injected mice testes (BTB). Our data confirmed that MBZ suppressed the expression of the BTB junction-associated proteins and disrupted the Sertoli cells’ function in vivo. Moreover, MBZ-treated mice demonstrated an aberrant caspase-3 signalling pathway, which resulted in the apoptosis of the germ cells. Here, we present our data, indicating that MBZ impairs BTB by reducing the expression of the microtubules’ and BTB junction-associated proteins. The last leads to activating the caspase-3 pathway, which triggers extensive germ cell apoptosis.

## 1. Introduction

Mebendazole (MBZ) is a well-tolerated and commonly-used benzimidazole antihelmintic agent. It can limit cell proliferation in parasitic worms by suppressing their tubulins [1]. As reported in many clinical trials, the use of MBZ has proven safe and effective in a wide range of differently-aged populations [2]. Moreover, data show that MBZ in 200 mg per day, accepted as the generally applied concentration, up to 50 mg/kg/d, applied at echinococcosis, was safe, too [3]. Besides its anthelmintic activity, numerous studies have demonstrated that MBZ can suppress different tumors [3,4,5,6,7]. The authors reported that upper MBZ doses were applied (up to 100 mg/kg/day) and administrated by injection in the peritoneum or orally [4,8]. Known as a low-cost standard medicine, as well as for its expressively operational capability in the disruption of tubulin [3,5], MBZ has attracted oncologists to add new dimensions, in terms of its medical application, namely as an anticancer drug [6,7]. Lately, the improvements in cancer therapies have increased survival, yet several side effects are observed in most chemotherapy drugs. Concretely, one glaring example is that chemotherapy works successfully on tumor cells and leads to severe damage to spermatogenesis, leading to deteriorated sperm count and semen quality [9,10]. Notably, flubendazole, another methylcarbamate benzimidazole antiparasitic drug, has a noticeable macrofilaricidal influence on many thread-like parasitic roundworms [11]. Interestingly, data showed that flubendazole, at a dose of 100 mg/kg/day, utilized to treat dogs, led to testicular degeneration and disorders in spermatogenesis [12]. The accumulated data is still insufficient to enable us to identify the MBZ-induced impact on male reproductive health.

Spermatogenesis is decisive for normal male reproductive health. It is a well-regulated biological process of male sex cells differentiation and production, which takes place in the epithelium of the seminiferous tubules. The seminiferous tubule is where the self-renewal of the spermatogonia stem cells (SSCs), meiosis of the spermatocytes, and production of mature spermatozoa happens [13]. This epithelium represents a unique testicular microenvironment, isolated by BTB, providing vital preconditioning for the generation of sperm cells, and it is essential for sustaining male reproductive function [14]. BTB exactly divides the epithelium into basal and apical compartments [15,16,17], in which the sperm germ cells migrate. Moreover, this process, as well as the discharge of mature sperm cells, entails appropriate cell connections. These cell connections in the testis are called ectoplasmic specializations (ESs); in mammals, they are distinguished as gap (GJs), tight (TJs), and basal junctions. The extensive and dynamic cytoskeletal network allows the Sertoli cells to nurture and mechanically maintain the differentiating germ cells [18]. The cytoskeleton of these nurturing cells comprises of plentiful microtubules (MTs), which build a cytoplasmic network of α- and β-tubulin heterodimers. These heterodimers have a specific orientation in lined groupings, parallel to the cellular long axis. They further form a longitudinally-oriented, cage-like structure surrounding the cell nucleus and protrude toward the desmosome junctions between them and the germ cells [19]. Any alterations in the Sertoli cells’ microtubule dynamic forces, by depolymerizing drugs, reduce the basal and apical cytoplasmic processes and affect sperm germ cells maturation [20].

Guerini et al. suggested that MBZ could be developed into a drug for malignant diseases [3]. Nevertheless, in the literature, there is insufficient proof to answer whether it affects the patients’ reproductive health and, if so, what the underlying mechanism of this action is. Therefore, our better understanding of the biochemical pathways that underlie the male fertility issue induced by MBZ is of great importance. The objective of our study is to explore these underlying mechanisms of MBZ sperm cells’ cytotoxicity, in order to assist in the development of new agents that will protect the male reproductive system from MBZ-induced damage. Our results provided an investigation on the short caspase-3 activation and disruption of microtubules in the testes of MBZ-injected mice. We further demonstrated that a single intraperitoneal injection of MBZ triggered transient functional disruption of BTB and cell apoptosis in the testes.

## 2. Results

### 2.1. Subsection

#### 2.1.1. Mebendazole Triggers Testicular Damage in Mice Testes

**Figure 1:**
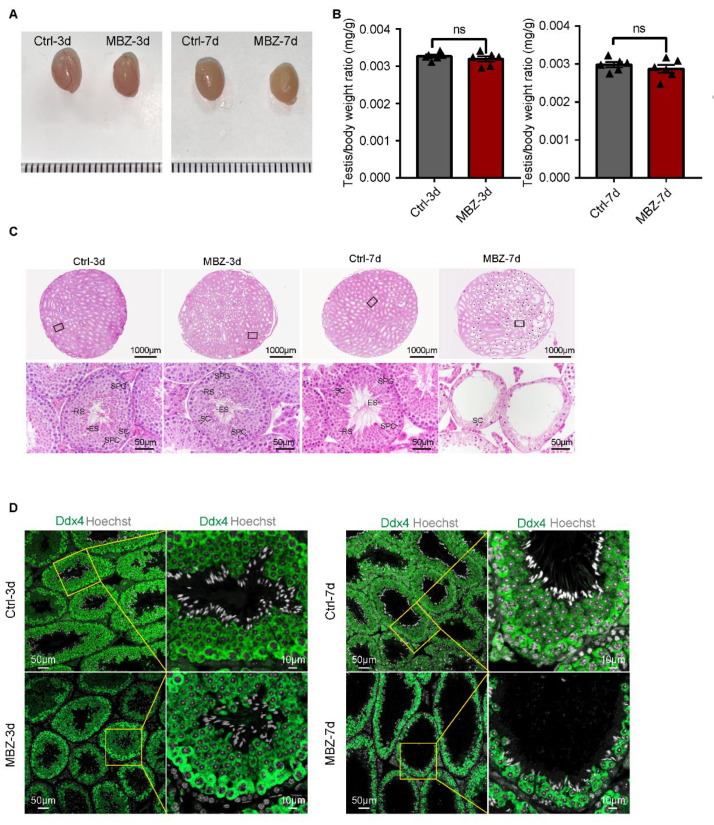
MBZ-treated mice exhibit an abnormal spermatogenic phase. (**A**) Images of testes from MBZ- and DMSO-injected (control) mice. (**B**) Normalized rations of the testes and bodyweight of the MBZ-treated and control mice. Data are represented as MEAN ± SEM, *n* = 6. Six sets of biological replicates were performed. The six values are indicated as six triangles on each bar. The results were considered non-significant at *p* > 0.05, assessed by *t*-test. (**C**) Testicular sections with HE-staining, where SPG means spermatogonia; SPC: spermatocyte; RS, round spermatids; ES: elongated spermatids; SC: sertoli cells; * asterisk designates damaged seminiferous tubule; scale bars = 1000 and 50 μm. (**D**) Testicular sections from the studied groups of mice, in which the germ cells are labelled with Ddx4. Cell nuclei were stained with Hoechst (grey). Scale bar = 10 μm.

After intraperitoneal injection, containing MBZ (40 mg/kg) or control physiological saline buffer with DMSO (1:1), the animals were euthanized at two time points: days 3 and 7, respectively. The defects in the testicular tissues were subsequently monitored. Gross examination of the size of the studied mice testes revealed no significant differences on the two days between the MBZ-treated and control mice groups (Figure 1A). Additionally, the comparison between the MBZ-treated and control groups did not demonstrate differences in the calculated ratios between the testes and body weights (Figure 1B). To analyze the effects of MBZ on spermatogenesis, we performed hematoxylin–eosin (HE) staining. The mice testes on the 3rd day after the injection showed normal spermatogenesis, similar to the control. However, for the group of MBZ-treated ones, on the 7th day, we detected untimely sloughing off of a large number of germ cells from the seminiferous epithelium, which led to noticeable thinning of the spermatogenic epithelium in more than half of the testicular lumen (Figure 1C). We then labeled the germ cells with Ddx4 by immunofluorescence (IF) and found that the proportion of Ddx4-positive cells in the group of MBZ-treated mice (euthanized after 3 days of the injection) did not showcase substantial alterations. Yet, the amount of Ddx4-positive cells, in mice treated with MBZ and euthanized on the 7th day after the injection, was considerably decreased, in comparison to the control group, which proved to be consistent with the obtained results from the HE-staining (Figure 1D and Figure S1).

**Figure 2:**
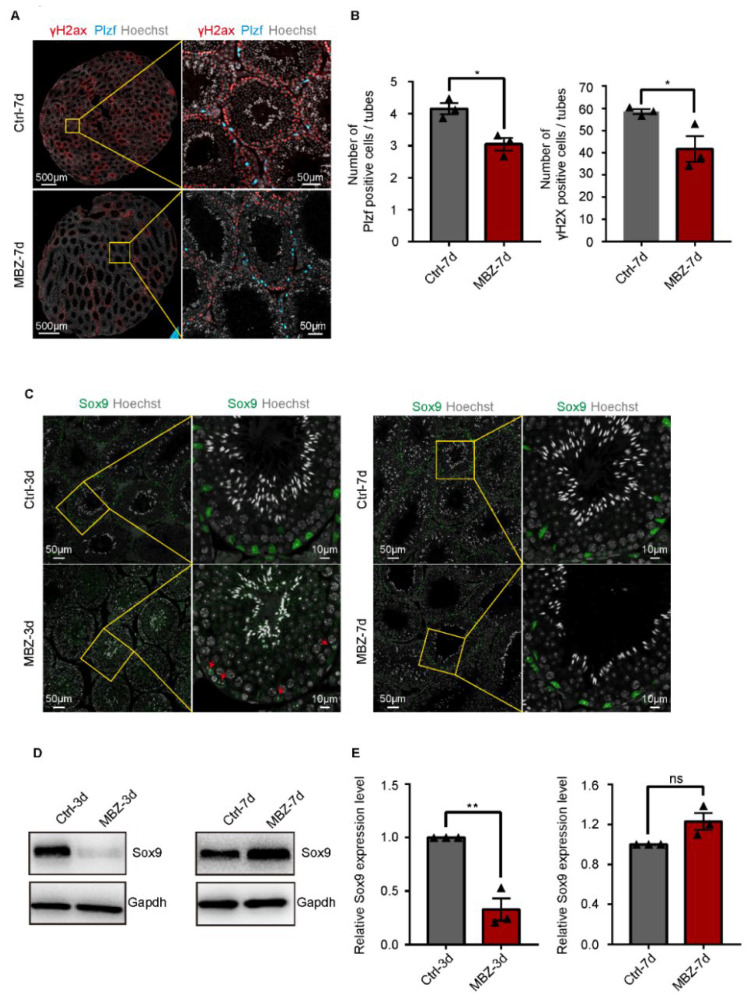
MBZ leads to a loss of germ cells and impaired function of Sertoli cells. (**A**) Co-IF staining of Plzf and γH2Ax in the testes of MBZ-treated and control mice on the 7th day post-injection; scale bars = 50 and 10 μm. (**B**) The average number of Plzf and γH2Ax positive cells, per tubule, in the testes of mice exposed to MBZ after the 7th day; (**C**) IF staining of Sox9 in the MBZ-treated and control testes. Cell nuclei were stained with Hoechst (grey); red arrow: Sertoli cell; scale bars = 50 and 10 μm. (**D**,**E**) Western blot analysis of Sox9 in the analyzed group of animals. GAPDH served as a normalization control. WB results were visualized by Image J. Data are MEAN ± SEM, *n* = 3. Three sets of biological replicates were performed. The three values are indicated as three triangles on each bar. The obtained results were statistically evaluated by the student’s *t*-test, where *p* values < 0.05 (*) and <0.01 (**) were considered statistically significant.

We further counted the number of spermatogonia and spermatocytes in the testes of mice after 7 days of the injection. The visualization was done by IF, after staining with anti-Plzf and anti-γH2ax antibodies to represent spermatogonia [21] and spermatocyte [22]. Interestingly, we observed a decrease in the average number of spermatocytes and spermatogonia per tubule in the testes of MBZ-treated mice on the 7th day, when compared with the control (Figure 2A,B). We then labeled the Sertoli cells with anti-Sox9 antibodies by IF [23]. Furthermore, Sox9 expression in the testes of MBZ-treated mice on the 3rd day was done and compared to the control. The obtained results showcased a decrease in the expression in MBZ-injected mice testes. On the contrary, when we compared Sox9 expression in the testes of MBZ-treated mice on the 7th day with the control, we received similar results (Figure 2C). Since the difference in Sox9 expression was observed by IF, we speculated that the Sertoli cells were damaged in the testes of mice on the 3rd day, after drug administration. As expected, the analysis by western blot (WB) showed that Sox9 expression in testes treated with MBZ was decreased after 3 days of the treatment, when compared to the control. This confirmed our suggestion that the Sertoli cell’s function was impaired on day 3 (Figure 2D,E and Figure S2A). On day 7, though, the expression levels of Sox9 in MBZ-treated testis were slightly higher than those in the control groups (Figure 2D,E and Figure S2B). Our data from these experiments indicate that MBZ destroys the function of mice Sertoli cells 3 days post-injection. It further disorders the phases of spermatogenesis and leads to a reduction in the number of germ cells within 7 days after treatment.

#### 2.1.2. BTB-Related Junction Proteins Exhibit Destruction in Mebendazole-Treated Mice

**Figure 3:**
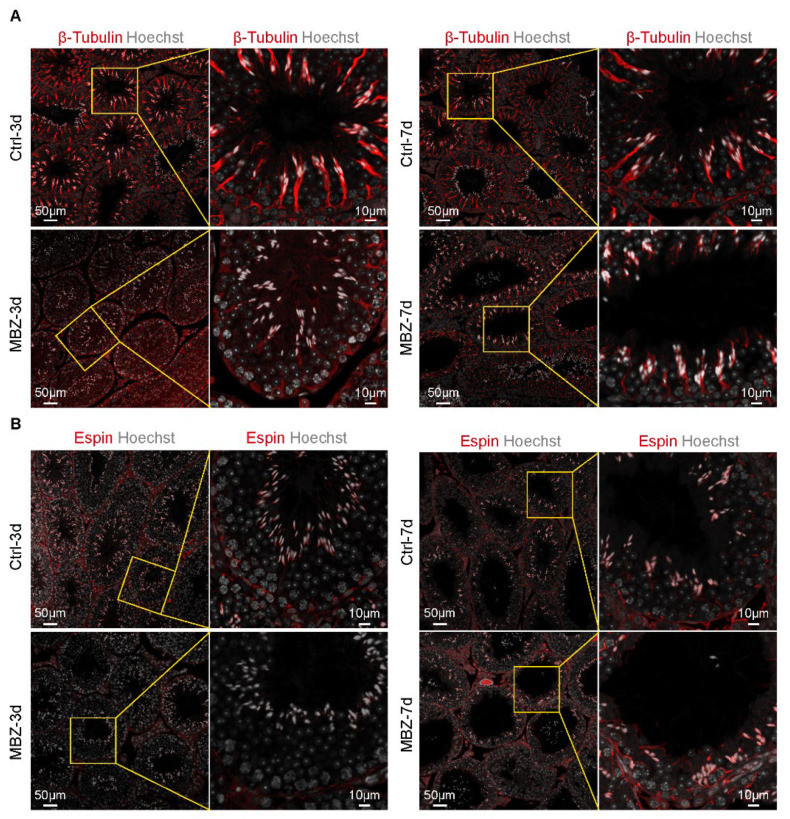
MBZ impacts the microtubule network of Sertoli cells. (**A**) Fluorescence staining of β-tubulin in the studied groups of animals. Scale bars = 50 and 10 μm. (**B**) Fluorescence staining of espin. Scale bars = 50 and 10 μm.

**Figure 4:**
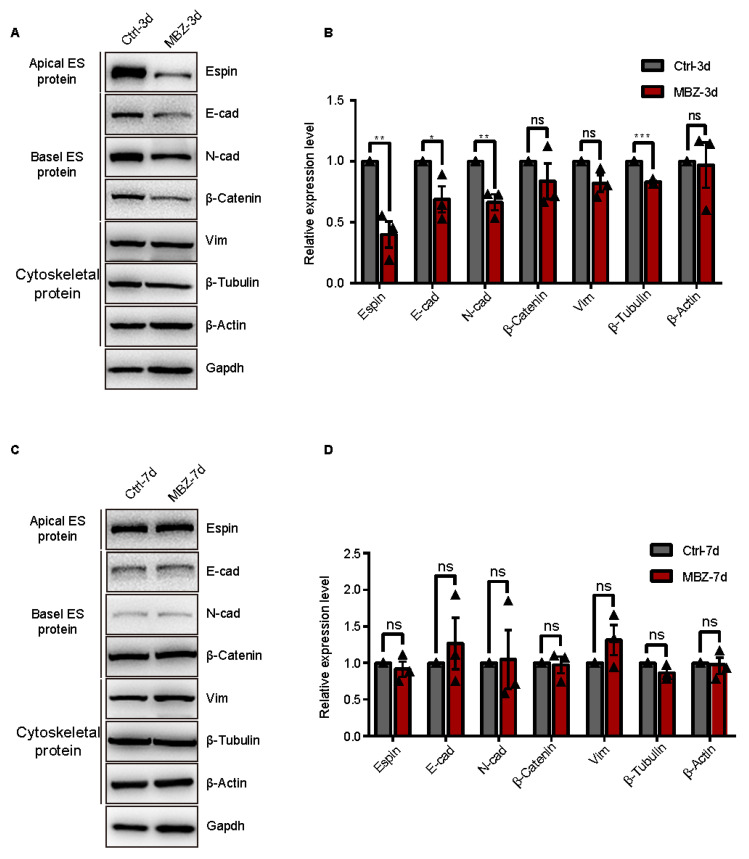
MBZ influences the BTB-related proteins. (**A**,**B**) Western blot analysis of BTB-related proteins in the testes of control and MBZ-injected mice, 3 days post-injection. GAPDH served as a normalization control. (**C**,**D**) WB of the BTB-related proteins in the testes of the two groups of animals on the 7th day after their exposure. GAPDH served as an internal control. Results were evaluated by Image J and are presented as MEAN ± SEM, *n* = 3. Three sets of biological replicates were performed. The three values are indicated as three triangles on each bar. Values that were non-significant had *p* > 0.05, while those that were significant had * *p* < 0. 05, ** *p* < 0.01, and *** *p* < 0.001.

MBZ is an inhibitor of tubulins [7], which represents a cytoskeleton serving as a structural supporter of the vesicles. It further potentiates organelle transport, chromosome replication separation, intracellular transport, cell movements, and polarization [24]. This cytoskeleton drives the testicular Sertoli and germ cells’ development [19]. We conducted IF staining to assess the expression and cellular distribution of β-tubulin in testicular tissue sections. The detected fluorescence signals in the control groups of animals were conspicuously located at the apical ES and BTB, as well as the β-tubulin rich ultra-structures at the Sertoli-spermatid and Sertoli cell–cell interface, respectively [25]. There were few high β-tubulin intensity in these ultra-structures and disordered locations in MBZ-treated testes on the 3rd day, which were recovered in the mice examined on the 7th day (Figure 3A). Furthermore, we performed IF to study the ES protein, espin. Espin is an actin-binding protein responsible for the dynamic remodeling of the actin bundles [25]. IF allowed us to evaluate its levels and location in the testes of the studied mice. Our results showed suppression of the espin expression on the 3rd day, after MBZ administration and recovery on the 7th day, concerning β-tubulin (Figure 3B). The findings indicate that MBZ disturbed the spreading of connection proteins and suppressed the cytoskeletal organization, further impacting the BTB. To verify this hypothesis, we conducted WB analyses for the BTB components, including the E-cad, N-cad, β-catenin (basal ES-related junction proteins), espin (apical ES-related junction proteins), VIM, β-actin, and β-tubulin (cytoskeletal proteins). The analyses showed that protein expressions of β-tubulin, espin, E-cad, and N-cad were significantly decreased, while β-catenin and VIM showed a trend to decrease. The expression of other cytoskeletal protein (β-actin) did not change in the MBZ-treated groups on the 3rd day, compared to the control (Figure 4A,B and Figure S2A). In MBZ-treated testes on the 7th day, the detected downward trend was reversed, and their protein levels were comparable to these of the control (Figure 4C,D and Figure S2B). These data propose that tubulin, after MBZ treatment, temporarily depolymerizes, leading to a suppression of BTB-related proteins’ levels in testes.

#### 2.1.3. Mebendazole Treatment Initiates Cell Apoptosis in Mice Testes and Epididymis

**Figure 5:**
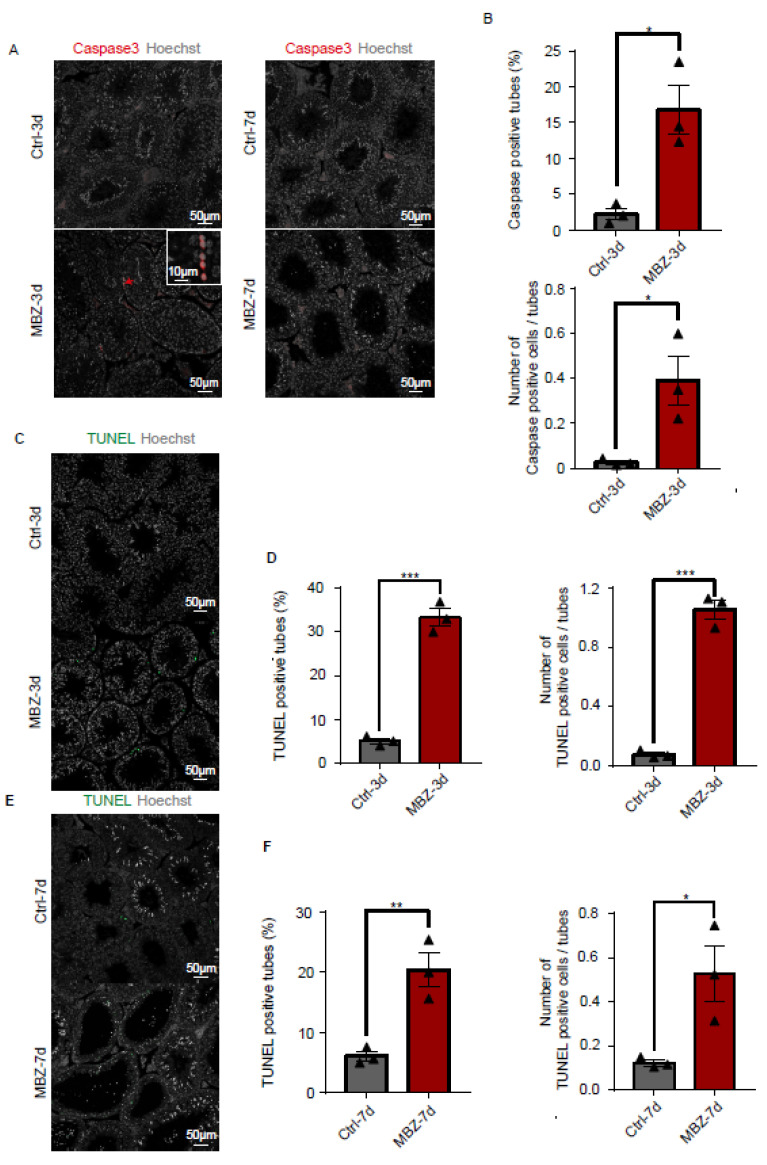
MBZ induces caspase-3 activation and apoptosis. (**A**) Fluorescence staining of caspase-3 in the testes of the studied groups of animals. Red arrow highlights signals of caspase-3; scale bar = 50 μm. (**B**) Caspase-3-positive tubules and quantitation of their average count per tubule in MBZ-treated testes on the 3rd day after their exposure to MBZ, as well as in control testes. (**C**) The effect of MBZ on cell apoptosis, detected by TUNEL staining on the 3rd day. Scale bar = 50 μm. (**D**) The TUNEL-positive tubules were expressed in percentage, as well as in average count per tubule in MBZ-treated testes on the 3rd day. (**E**) The effect of MBZ on cell apoptosis by TUNEL staining on the 7th day post-injection. Scale bar = 50 μm. (**F**) Percentage of TUNEL-positive tubules and average count per tubule in MBZ treated on the 7th day. Results are given as MEAN ± SEM, *n* = 3. Three sets of biological replicates were performed. The three values are indicated as three triangles on each bar. They were considered statistically significant when *p* values were <0.001 (***), <0.01 (**), and <0.05 (*).

The above-presented results showed that the injection of MBZ in mice caused severe testicular destructions. Given that MBZ has been reported to activate the caspase-3 signalling pathway and induce apoptosis [3], we initially evaluated the number of caspase-3-positive cells in the testes of the studied groups of animals by IF staining. Our data showed a significant increase in caspase-3-positive cells in the MBZ-injected mice on the 3rd day post-injection. On day 7, no significant increase in caspase-3-positive cells number was observed in the injected mice, compared to the control (Figure 5A,B). We further performed TUNEL staining on the testes. The results indicated a substantial escalation in the number of cells undergoing apoptosis in the MBZ treated on the 3rd day post-injection (Figure 5C,D). In comparison, the TUNEL apoptotic cells had a few additions in the 7th-day MBZ-treated testes, compared to control groups (Figure 5E,F). These results suggested that MBZ-induced apoptosis by activating of the caspase-3 s pathway, which was another reason for the observed impaired testicular function.

#### 2.1.4. Mebendazole Leads to Significant Abnormalities in the Sperm Cells in Mice Epididymis

**Figure 6:**
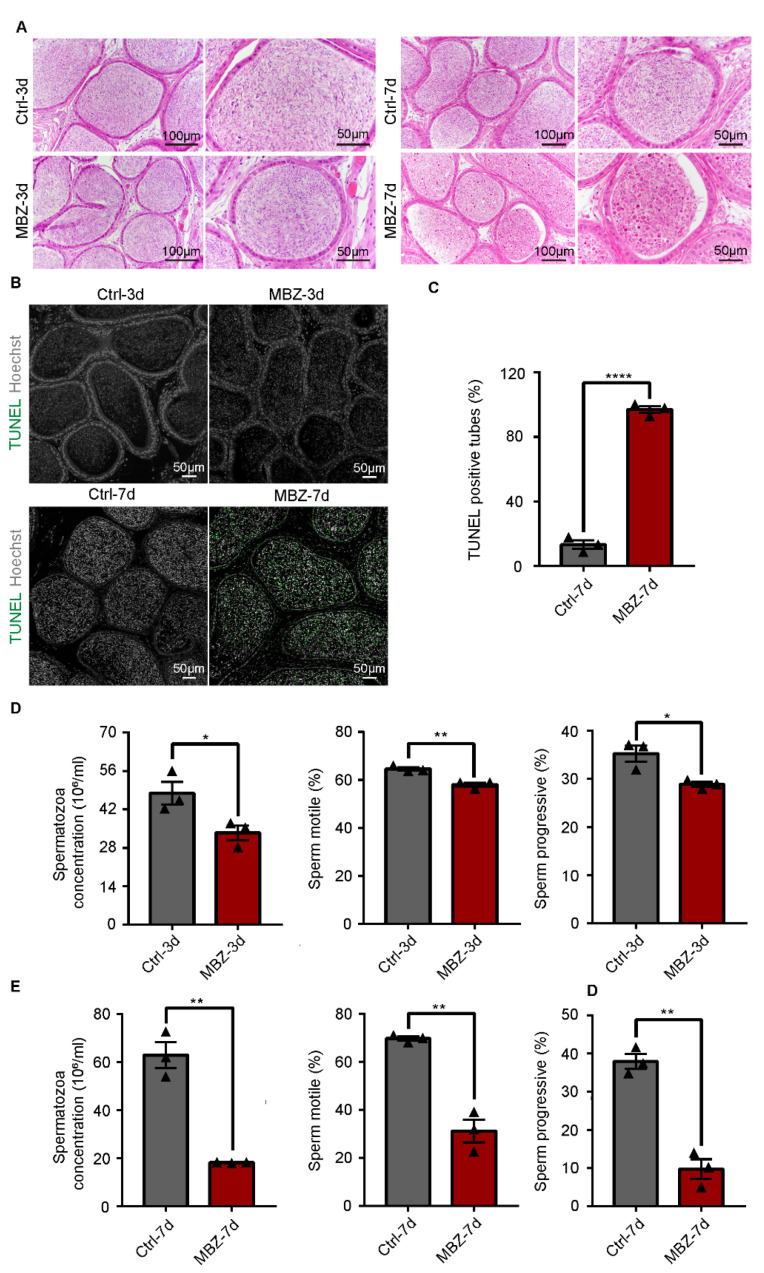
Sperm motility is impaired in mice treated with MBZ. (**A**) HE-stained sections of cauda epididymis from control and MBZ-treated mice. Scale bars = 100 and 50 μm. (**B**) TUNEL staining of cauda epididymis from control and MBZ-treated mice. Scale bar = 50 μm. (**C**) Percentage of TUNEL-positive tubules in MBZ-treated and control mice on the 7th day post-injection. (**D**,**E**) Cauda epididymal sperm contents, average frequencies of motile sperm on the 3rd and 7th day post-MBZ injection. Data are presented as MEAN ± SEM, *n* = 3. Three sets of biological replicates were performed. The three values are indicated as three triangles on each bar. Results were considered statistically different when *p* values were <0.0001 (****), <0.01 (**), and <0.05 (*).

**Figure 7:**
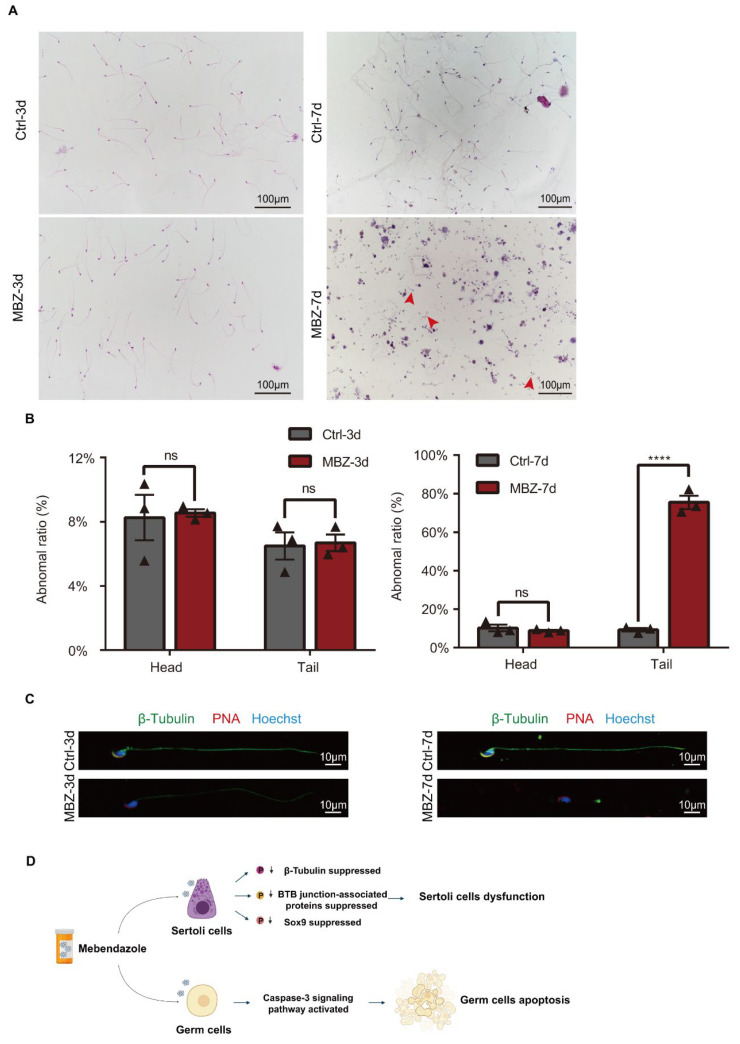
Teratozoospermia is obvious in MBZ-treated mice. (**A**) HE-stained spermatozoa from control and MBZ-treated mice. Red arrows highlight sperm with tail malformations; red arrow: tail deformities of sperm; scale bar = 100 μm. (**B**) Sperm with abnormal morphology are presented as a percentage. Data are given as MEAN ± SEM, *n* = 3. Three sets of biological replicates were performed. The three values are indicated as three triangles on each bar. (**C**) β-tubulin and PNA IF detection in control and MBZ-treated spermatozoa. Scale bar = 10 μm. Results were considered statistically different when *p* values were <0.0001 (****). (**D**) Schematic diagram shows the potential mechanisms of mebendazole-induced injury in mice testes.

Few adverse influences could be observed on the epididymis cauda, when operated H&E-staining and TUNEL analysis from these two groups of mice 3 days after intraperitoneal injection (Figure 6A,B). Alternatively, in the testes of mice on the 7th day after the administration, the epididymal sperm proved to be highly abnormal (Figure 6A). TUNEL analysis of epididymis sections showed a large number of apoptotic sperm in the 7th-day group (Figure 6B,C). Therefore, we respectively evaluated the sperm quality in MBZ-treated mice and control groups on the two observed time points, as in the previous experiments by a computer analyzer (CASA). Our results showed a slight difference in the overall epididymal cauda sperm concentration and motility between the MBZ and control group of mice 3 days after treatment (Figure 6D). Nevertheless, the comparison between the MBZ-treated mice on the 7th day post-MBZ injection with the control group showed that the sperm motile rate was decreased from 69.6% to 31.2%, while the progressive motile rate was decreased from 37.9% to 9.7%, on average, in the MBZ-injected animals (Figure 6E). Notably, the sperm concentration in the MBZ-treated group fell from 63.9 × 10^6^ to 18.3 × 10^6^/mL. The recorded data also confirmed the morphological abnormality of the sperm, as HE-staining revealed, with abnormalities in both sperm tails and heads, more concretely, 76% ± 6% and 8.4% ± 1.5%, respectively. In contrast, the comparison between the control and MBZ-treated mice, after 7 days of the injection, demonstrated that head or tail defects were found to be in only 9% ± 1.5% and 10.8% ± 1.5% of the sperm, but nearly no difference was detected in mice exposed to MBZ after 3 days. (Figure 7A,B). Interestingly, the immunofluorescent staining of the PNA (to represent acrosome of sperm) and anti-β-tubulin antibody demonstrated a major malformation of the sperm tail in the MBZ treated on the 7th day post-injection (Figure 7C). We also observed that the fluorescence intensity of β-tubulin declined in the MBZ-treated groups, which might explain the malformation of sperm (Figure 7C). The sperm density of the MBZ-treated mice decreased to nearly one-quarter, while an increase of sperm deformity was fourfold. Tail deformities were considered the most common defect.

## 3. Discussion

We examined the change of testicular histomorphology through HE-staining and found that contraction deformation of seminiferous tubules and a reduction of germ cells in the lumen were present in the MBZ-injected mice on the 7th day. Meanwhile, we assessed the histo-morphology of cauda epididymidis and analyzed sperm from cauda epididymidis by CASA and HE-staining. We found a significant reduction in the quality and quantity of sperm, accompanied with massive apoptosis cells, which presented as more extent eosinophilic staining in the MBZ-injected mice on the 7th day. Thimon et al. reported that the sperm cells are transferred from the testes to cauda epididymidis, which lasts up to 10 days in mice [26]. Epididymal transit is when the sperm acquires progressive motility and gains fertilizing ability [26]. We observed considerable amounts of apoptotic signals in the cauda epididymis of mice treated with MBZ for 7 days. Still, only a small number of apoptotic cells in the testis of mice were treated with MBZ for 3 days. Apoptotic sperm in the testis could not be transported to the cauda epididymis within 4 days. Therefore, we consider that MBZ also causes certain damage to the epididymis. These findings suggested that MBZ caused testicular and epididymidis toxicity in mice. Other tubulin-binding agents (TBAs), such as albendazole and flubendazole, are anti-anthelmintic compounds that have also shown testicular and epididymal toxicity. Albendazole-treated mice showed testicular hypoplasia after receiving 400 mg/kg/day for 104 weeks. Albendazole is a teratogenic agent with fetal toxicity to experimental animals. Flubendazole-treated rats showed testicular and germ cell degeneration, as well as luminal cell debris and reduced luminal sperm in the epididymides. Few testes damage mechanisms have been mentioned in these previous studies [12,27].

The inhibitor of the apoptosis proteins (IAPs) family plays a significant role in inhibiting caspases [28]. Overexpression of IAPs is prevalent in many types of human tumors and has been associated with chemoresistance, disease progression, and poor prognosis [29]. Inducing the expression of testis-specific IAPs may be the critical point to reduce the testis injury from MBZ treatment in cancer therapy.

We considered that a large number of shedding germ cells in MBZ-treated testes might be associated with tissue inflammation stimulation and NLRP3 inflammasome activation. Overactivation of NLRP3 inflammasome can cause germ cells reduction and spermatogenesis disruption [30,31]. Adenosine A2A receptor agonist PDRN and anti-inflammatory trace element selenium contribute to treating NLRP3 inflammasome-induced testicular injury [32].

Another study has revealed that MBZ can bind to the MTs plus ends, thus inhibiting tubulin polymerization and preventing the adding of tubulin subunits, which, finally, leads to apoptosis by activating the caspase-3 signalling pathway [33]. Disturbance of the Sertoli cells’ microtubule forces results in less basal and apical cytoplasmic processes related to germ cells [20]. This leads to sloughing between different germ cell cohorts with elongating spermatids, in particular [34]. In our study, the MBZ treatment disrupted the distribution and expression of MTs, led to apoptosis, and decreased the protein levels of BTB-related junction proteins 3 days post-injection, which were recovered on the 7th day. These findings indicated that a single intraperitoneal injection of MBZ caused a transiently compromised BTB function and temporary imbalance of the dynamic network of MTs. Moreover, MBZ induces a dose-dependent cell cycle block at the G2/M phase [5,6,33]. MBZ also inhibits MAPK pathway activation by inhibiting phosphorylation of ERK1/2, which is related to cell proliferation [35]. We observed a decreased number of spermatogonia cells in mice treated with MBZ for 7 days. Therefore, we hypothesized that MBZ might impact the self-renewal of SSCs, yet we have no evidence to prove it. Taking into consideration the stages of clinical trials for the treatment of cancer, we have enough reasons to believe that a long-term MBZ-treatment will induce a large amount of apoptosis and directly influence spermiogenesis. Though, additional investigations are required to clarify the influence of MBZ therapy on reproductive health.

A crucial protein in the genesis of testes in mice is Sox9 [36,37]. Other authors have proved that Sox9 connects to MTs and destabilizes them [38]. The last abolishes the nuclear translocation of Sox9 proteins [39], whereas the letdown of Sox9 to come in the nucleus leads to gonadal dysgenesis [40] and XY sex reversal [41]. Sox9 interacts with the MTs network, as suggested by Malki and colleagues [38]. Concerning the MT cytoskeleton of the Sertoli cell, it experiences remodeling through embryonic phases to maintain the development of testis assembly [38]. Other authors revealed that oestrogen can activate ERK1/2 to mediate the stabilization of MTs and, thus, lead to the preservation of Sox9 in the cytoplasm [42]. One of the MTs’ functions is to reorganize spermatogenesis regularly to facilitate the remodeling of the Sertoli cell’s form, which is obligatory for the provision of the germ cells in adult animals [43]. Our investigation indicated that the expression of Sox9 was certainly connected with the stability of MTs in mature Sertoli cells in testes. To the best of our knowledge, this is the first investigation on the matter. It is of significant importance, though, to clarify the underlying machinery of all these reported findings exploring the role of Sox9 in the regulatory network for the control of MTs in mature testes. 

In summary, we identified that MBZ had severe toxic effects on the male reproductive system. MBZ induced extensive germ cell apoptosis by activating caspase-3. Moreover, our results gave an understanding of MT participation in testes damage, particularly concerning BTB injury, which allowed us to enhance the investigation of possible molecular mechanisms of MBZ-induced reproductive toxicity and possibly novel potential targets for preventive and therapeutic treatments (Figure 7D).

## 4. Materials and Methods

### 4.1. Animal and Treatment

C57BL/6 (B6) mice were purchased from the Jiangsu Animal Experimental for Medical Pharmaceutical Research Center. All animal-involving procedures were done following the ethical rules of the Nanjing Medical University. The experimental protocol was permitted by the Nanjing Medical University Institutional Animal Care and Use Committee (IACUC) (protocol number: 2009002). All mice were kept under SPF environments (12 h light/dark cycle, T °C: 22–26 °C, under 50–70% humidity), employing sufficient water and food supply. All our experiments were performed according to ARRIVE guidelines.

The animals were administered 40 mg/kg of MBZ by a single intraperitoneal injection. The control group was given the same volume of physiological saline buffer with dimethyl sulfoxide (1:1). The animals were euthanized; they were examined on the 3rd and 7th days, post-injection (n = 3).

### 4.2. Histological Analysis

The tissues were fixed in 4% paraformaldehyde (4%PFA) or modified Davidson’s fluid solution (mDF) for 48 h at 4 °C and sequentially washed in 70, 80, 90, and 100% ethanol. Washing in xylene:ethanol (1:1) and 100% xylene followed. The tissues were fixed in paraffin, while the cut sections for histology were 5 μm thick. HE-staining was performed using a hematoxylin (G1005, Servicebio, Wuhan, China) and eosin staining solution (E607321, Sangon Biotech, Shanghai, China). IF was performed by dewaxing and rehydrating sections, which were then heat-mediated for antigen recovery in 10 mM NaCl solution (pH 6). Sections were submerged in 5% bovine serum albumin (5% BSA; B0012, SunshineBio, Nanjing, China) and, after that, were incubated with the primary antibodies at 4 °C ON (overnight). The sections were then washed in PBS and later incubated with Alexa fluorophore (488, 594, and 647)-conjugated secondary antibodies (Thermo Fischer Scientific, Waltham, MA, USA) at room temperature (RT °C) for 2 h in the dark. The primary/secondary antibodies were diluted in 5% BSA. Sperm cells nuclear structures were stained with Hoechst 33342 (H3570, Thermo Fisher Scientific, Waltham, MA, USA), at a dilution of 1:1000 for 10 min, before being covered by glass coverslips and fixed with glycerol. TUNEL assay (A111-03, Vazyme, Nanjing, China) was used to detect testicular cells undergoing apoptosis, following the manufacturer’s protocol. Results were visualized under a laser scanning confocal microscope LSM800 (Carl Zeiss, Oberkochen, Germany). 

### 4.3. Epididymal Sperm Analysis

The right epididymis was stripped out; then, the cauda epididymis was dissected and suspended in HTF medium, supplemented with 10% FBS (90126, Irvine Scientific, Santa Ana, CA, USA), at 37 °C for 5 min. After 10 min incubation, the 10-µL sperm aliquots were examined by computer-assisted semen analysis (Hamilton Thorne Research Inc. Beverly, MA, USA). Specifically, we measured the motility and sperm concentration. At least 200 cells per sample were investigated. The sperm cells were spread on glass slides. Fixation in 4% PFA at RT °C followed for 40 min, after which HE-staining and morphological observations were performed.

### 4.4. Protein Isolation and Western Blot

Total testicular proteins were extracted by 8 M buffer (50 mM Tris–HCl pH 8.2, 75 Mm NaCl, 8 M urea), comprising of a protease inhibitor cocktail (B14002, Bimake, Houston, TX, USA). The protein concentrations were quantified by Bradford protein assay kit (P0006, Beyotime, Shanghai, China). Equivalent proteins were parted on 12% SDS-PAGE gel and blotted on polyvinylidene difluoride membrane (PVDF). Inhibition of unspecified antigens, with 5% non-fat milk dissolved in TBS, followed at RT °C for 2 h; then, the diluted primary antibodies were incubated at 4 °C ON. Washing the membrane with TBST (Tris-buffered saline with Tween) every 15 min for 3 times followed. Horseradish peroxidase (HRP)-conjugated secondary antibodies were crossed with the membranes for 2 h at RT °C. Finally, the signals were detected by chemiluminescence solution ECL. ChemiDoc XRS + System (Bio-Rad, Hercules, CA, USA). 

### 4.5. Antibodies

The first antibody against Sox9 for WB and IF was purchased from Invitrogen (AB5535, Millipore, Billerica, MA); anti-espin for WB and IF (611656, BD Biosciences, New York City, NY, USA); anti-β-tubulin for WB and IF (AB0039, Abways, Beijing, China); anti-E-cad for WB (3195, CST, Boston, MA, USA); anti-N-cad for WB (NBP1-48309,Novus Biologicals, Littleton, CO, USA); anti-β-catenin for WB (610153, BD Biosciences, New York City, NY, USA); anti-VIM for WB (AF2105, RD,Minneapolis, MN, USA); anti-β-actin for WB (ab8226, Abcam, Cambridge, UK); anti- GAPDH for WB (Abcam, ab9485, Cambridge, UK); anti-Ddx4 for IF (ab13840, Abcam, Cambridge, UK); anti-Plzf for IF (AF2944, RD, Minneapolis, MN, USA); anti-γH2AX for IF (ab26350, Abcam, Cambridge, UK).

### 4.6. Statistical Analysis

Data from the IF and Western blot assays were statistically analyzed by the independent Student’s *t*-test using GraphPad Prism 7 (GraphPad Software, Inc. San Diego, CA, USA). All experiments were repeated three times and represented as mean ± SEM.

### 4.7. Quantitative Analysis of Immunofluorescence Staining Sections

The three stained whole sections from control or MBZ-treated testes were observed, respectively, to count the total number of seminiferous tubules, total number of Plzf positive spermatogonia cells, and γH2Ax positive spermatocytes in each testis section manually. The total number of positive cells/seminiferous tubules gives the average number of positive cells per tubule in the testes.

The total number of TUNEL-positive or caspase-3-positive tubules and cells of three stained whole sections from control or MBZ-treated testes was counted manually. All seminiferous tubules within each testis section were counted. As long as the tubule contains at least one TUNEL or caspase-3-positive cell, it is regarded as a positive tubule. The positive tubules/total number of tubules shows the percentage of TUNEL-positive or caspase-3-positive tubules in mouse testes. The positive cells/total number of tubules shows the average number of TUNEL-positive or caspase-3-positive cells in each tubule.

### 4.8. Quantitative Analysis of Sperm Abnormalities

The control or MBZ-treated mouse sperm made for HE-staining were used for calculating morphological abnormality. About 200 sperm were counted in different fields, and the percentage of abnormal sperm was calculated as follows: the number of abnormal head or tail/total sperm counted equals the percentage of head or tail abnormalities.

## Data Availability

Not applicable.

## Supplementary Materials

The following supporting information can be downloaded at: https://www.mdpi.com/article/10.3390/ijms23084220/s1.
