# Supplementary material for: Mebendazole-Induced Blood-Testis Barrier Injury in Mice Testes by Disrupting Microtubules in Addition to Triggering Programmed Cell Death
Source: Int J Mol Sci. 2022 Apr 11;23(8):4220. doi: 10.3390/ijms23084220 (PMC9029725; doi:10.3390/ijms23084220)
Supplement: Supplementary file 1 [file ijms-23-04220-s001.zip › ijms-1664773-supplementary.pdf]

Supplementary Figure S1

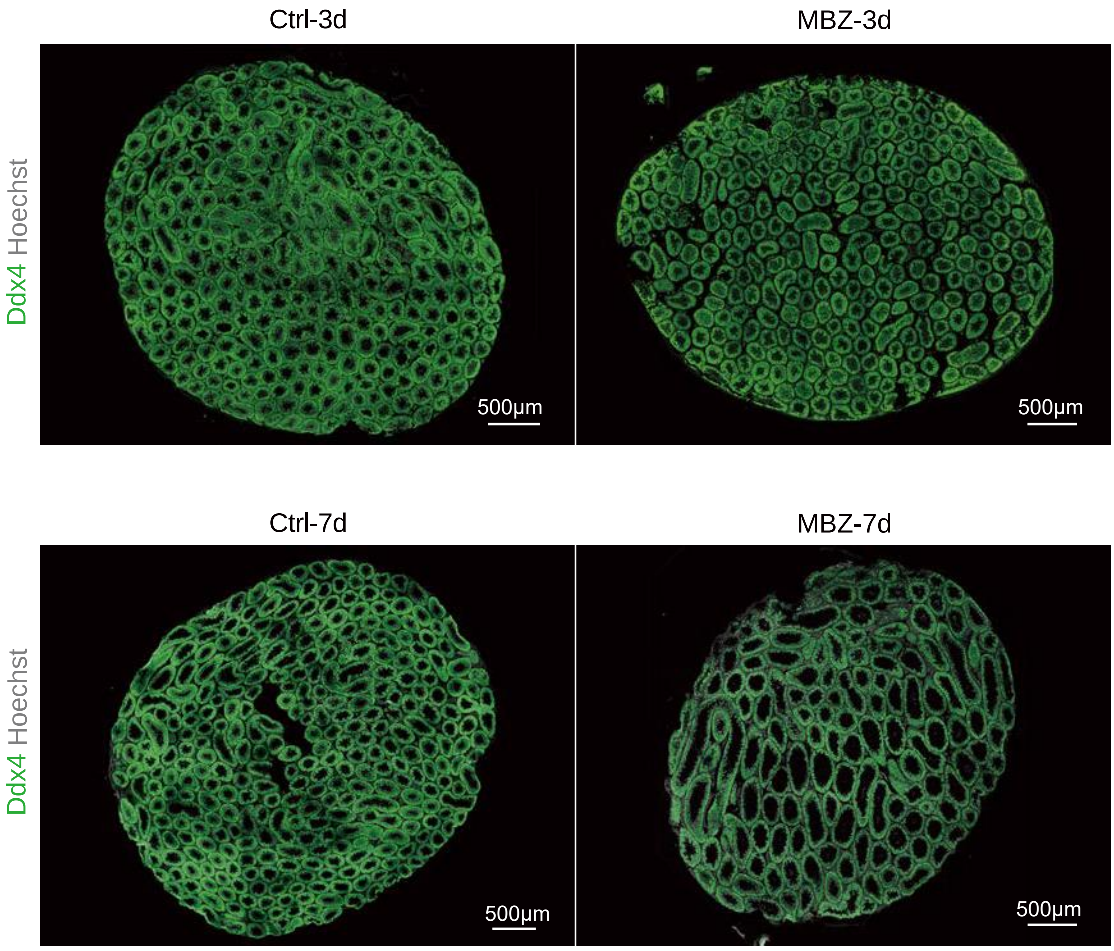

Figure S1. IF staining of Ddx4 in control and MBZ-treated mice.

Whole testicular sections from the studied groups of mice in which the germ cells are labeled with Ddx4. Cell nuclei were stained with Hoechst (grey). Scale bar = 500μm.

Supplementary Figure S2

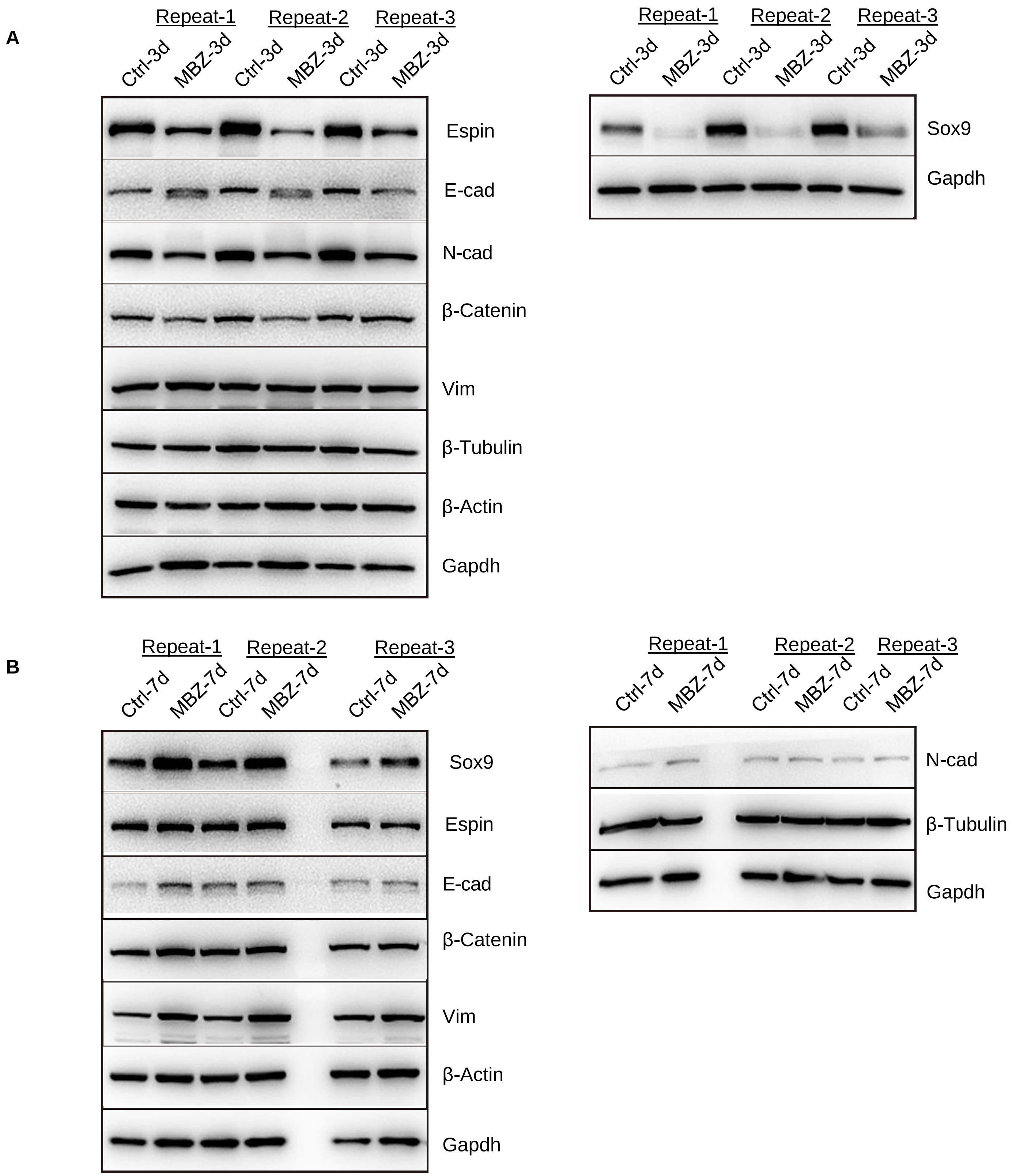

Figure S2. Western blot analysis of control and MBZ-treated mice testes.

(A) Western blot analysis of Sox9, β-Actin, β-Tubulin, Vim, Gapdh, N-cad, E-cad, β-Catenin, and Espin in the control testes and MBZ-treated testes exposed to MBZ after the 3rd day. GAPDH served as an internal control, n=3.

(B) Western blot analysis of Sox9, β-Actin, β-Tubulin, Vim, Gapdh, N-cad, E-cad, β-Catenin, and Espin in the control testes and MBZ-treated testes exposed to MBZ after the 7th day. GAPDH served as an internal control, n=3.
